# Retrospective review of superficial femoral artery stenting in diabetic patients: thiazolidinedione use may decrease reinterventions

**DOI:** 10.1186/1471-2261-14-184

**Published:** 2014-12-11

**Authors:** Karen L Walker, Daniel B Walsh, Philip P Goodney, Samantha A Connell, David H Stone, Richard J Powell, Eva M Rzucidlo

**Affiliations:** Section of Vascular Surgery, Dartmouth Hitchcock Medical Center, 1 Medical Center Drive, Lebanon, NH 03756 USA

**Keywords:** Adiponectin, Diabetes, Peripheral arterial disease, Thiazolidinediones, Endovascular

## Abstract

**Background:**

Diabetics are known to have inferior outcomes following peripheral vascular interventions. Thiazolidinediones are oral diabetic agents which improve outcomes following coronary bare metal stenting. No studies have been performed evaluating thiazolidinedione use and outcomes following lower extremity endovascular interventions. We hypothesize that diabetic patients taking thiazolidinediones at the time of primary superficial femoral artery (SFA) stenting have fewer reinterventions.

**Methods:**

A retrospective review was performed to identify diabetic patients undergoing primary SFA stenting. The unit of analysis was the extremity. The primary outcome was freedom from target lesion revascularization stratified by thiazolidinedione use, evaluated by Kaplan Meier curves and a log rank test. A Cox proportional hazards model was constructed to determine variables associated with freedom from target lesion revascularization.

**Results:**

SFA stents were placed in 138 extremities in 128 diabetic patients between August 1, 2001 and July 15, 2012. Twenty-four patients were taking thiazolidinediones at the time of SFA stenting. All patients taking thiazolidinediones had TASC A or B lesions. Twenty-seven extremities in the non-thiazolidinedione group had TASC C or D lesions and were excluded to control for disease severity. Freedom from target lesion revascularization was significantly higher in diabetics taking thiazolidinediones at 2 years, 88.5% vs. 59.4%, P = 0.02, SE < 10%. Cox modeling identified a protective trend for thiazolidinedione use (thiazolidinedione use HR 0.33, 95% CI 0.09-1.13), whereas critical limb ischemia and insulin use were associated with trends for worse freedom from target lesion revascularization.

**Conclusions:**

This pilot, translation study demonstrates that diabetic patients taking thiazolidinediones at the time of primary SFA stenting have decreased reintervention rates at 2 years. These results may be explained by higher adiponectin levels or other anti-inflammatory effects in patients taking thiazolidinedione. National and regional quality improvement registries should consider collecting information regarding specific diabetic regimens and use of PPAR agonists such as cilostazol and fibrates.

## Background

Superficial femoral artery (SFA) stenting has revolutionized the care provided to patients with lower extremity peripheral artery disease and is a commonly performed procedure [1]. Diabetes is a known risk factor for poor clinical outcomes, such as recurrent claudication, decrease in ankle-brachial index or failure to heal a wound [1–4]. Poor clinical outcomes following SFA stenting frequently result in reintervention to correct in-stent restenosis, narrowing of the stented arterial segment.

The reasons why diabetics are at increased risk for developing in-stent restenosis are largely unknown. However, studying clinical outcomes associated with specific medication regimens may prove useful. For example, prior studies have identified that use of exogenous insulin is a risk factor for restenosis following endovascular intervention in the coronary and peripheral circulations [2, 5, 6]. Alternatively, use of thiazolidinediones (TZDs) has been associated with decreased rates of in-stent restenosis for coronary bare metal stents [7–9].

While the association between TZD use and outcomes has been studied for patients undergoing coronary interventions, there is no data available for patients undergoing interventions for low extremity peripheral arterial disease. Therefore, we studied the association between TZD use and reintervention rate for diabetic patients undergoing SFA stenting. Our results demonstrate that the rate of reintervention was significantly lower in diabetics taking TZDs versus diabetics not taking TZDs at 2 years, 88.5% vs. 59.4%, P = 0.02, SE < 10%.

## Methods

**Figure 1:**
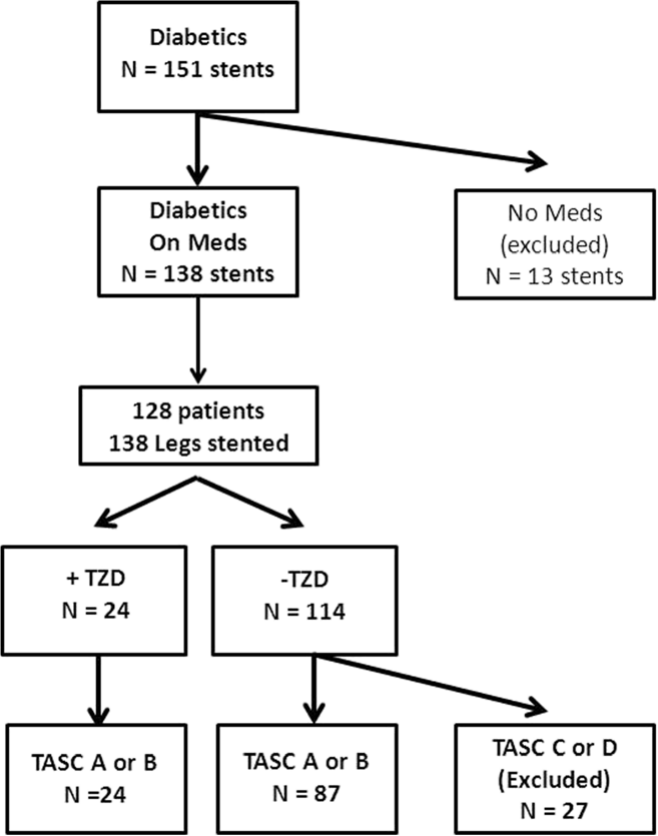
**Cohort description.** Cohort exclusion criteria are depicted.

We searched the vascular surgery database for elective SFA stents between August 1, 2001 and August 15, 2012 [3]. The analysis includes only diabetics undergoing primary interventions. Diabetics who were not taking medications for glycemic control were excluded from our analysis (n = 13) (Figure 1).

Diabetic patients were stratified by TZD use. Freedom from target lesion revascularization (TLR) was evaluated. After placement of an SFA stent, patients were evaluated at 1, 6, and 12 months and then yearly. Recurrent symptoms, decreased ankle brachial index, or a stenosis identified by arterial duplex with greater than a 2.5× step-up in peak systolic velocity prompted further investigation.

Patient demographics, comorbidities, and peripheral arterial disease severity characteristics were analyzed using Student’s t-test, chi square, and Fisher’s exact test. Chronic kidney disease was defined as creatinine > 1.6. Kaplan Meier survival curves using the peto method were created to test for differences in freedom from TLR.

A Cox proportional hazards model was created to identify predictors of freedom from TLR. Variables with a P < 0.2 in univariate testing were included in a Cox proportional hazards model, and backwards stepwise elimination was used to remove non-significant variables. The statistical analysis was performed using STATA 12.1.

The Committee for the Protection of Human Subjects at our institution granted institutional review board approval for this study.

## Results

**Figure 2:**
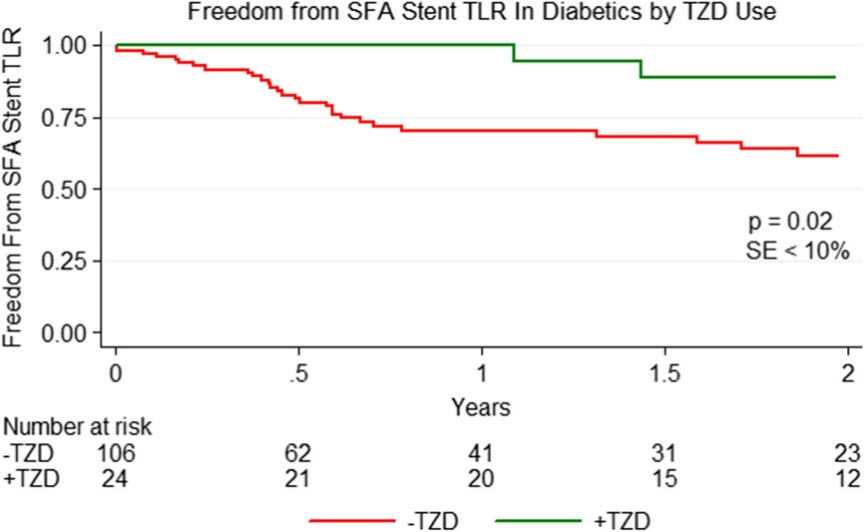
**Freedom from SFA Stent TLR among all diabetics by TZD use.** Freedom from TLR was 88.5% for diabetics taking thiazolidinediones at the time of SFA stenting vs. 61.2% for those not taking a thiazolidinedione at 2 years. This difference was statistically significant, p = 0.02, with a standard error < 10% at all points on the graph.

Between August 1st 2001 and July 15th 2012, the vascular surgeons at our institution performed 363 primary SFA stenting procedures. Of these, 128 diabetic patients who were taking diabetic medications underwent SFA stenting in 138 extremities (Figure 1). Demographics, comorbidities, and cardiovascular medication use were similar regardless of TZD use. Use of insulin, metformin, and sulfonylureas were also similar. However, significant differences related to disease severity variables were present (Table 1). Specifically, all patients taking TZDs had either a TASC A or B lesion and the prevalence of critical limb ischemia (CLI) was significantly lower among the TZD group. Technical success was achieved in all cases. The mean stented vessel diameter was 5.4 mm (95% CI: 5.3-5.5 mm) for patients not taking TZDs vs. 5.5 mm (95% CI: 5.2-5.8 mm) for patients taking TZDs, p = 0.39. The mean stented length was 15.2 cm (95% CI: 13.5-17.0 cm) for patients not taking TZDs vs. 8.9 cm (95% CI: 6.6-11.2 cm) for patients taking TZDs, p < 0.01. Stents grafts were used in 2 cases; these patients were not taking TZDs. Freedom from TLR was significantly higher for diabetics taking TZDs at 2 years, 88.5% vs. 61.2%, p = 0.02 (Figure 2).

**Table 1:** **Characteristics of diabetic patients undergoing SFA stenting**

	+TXD N = 24	-TZD N = 114	P-value
Age	64.46	69.57	0.06
Male	79.17%	54.39%	**0.03**
HTN	91.67%	88.60%	0.66
HLD	79.17%	81.58%	0.78
CAD	54.17%	54.39%	0.98
CKD	13.39%	14.91%	0.76
COPD	4.17%	11.93%	0.26
Current smoker	12.50%	22.81%	0.26
Statin	62.50%	69.03%	0.53
ASA	78.26%	79.44%	0.9
Plavix	39.13%	51.40%	0.29
Cilostazol	8.70%	14.02%	0.49
Insulin	50.00%	64.04%	0.24
Metformin	37.50%	35.09%	0.82
Sulfonylurea	29.17%	37.72%	0.49
CLI	33.33%	62.2/8%	**0.01**
TASC A or B	100%	76.32%	**0.01**
Preop toe pressure	52.5	40.95	0.18
3 Vessel outflow	41.67%	35.09%	0.54

**Figure 3:**
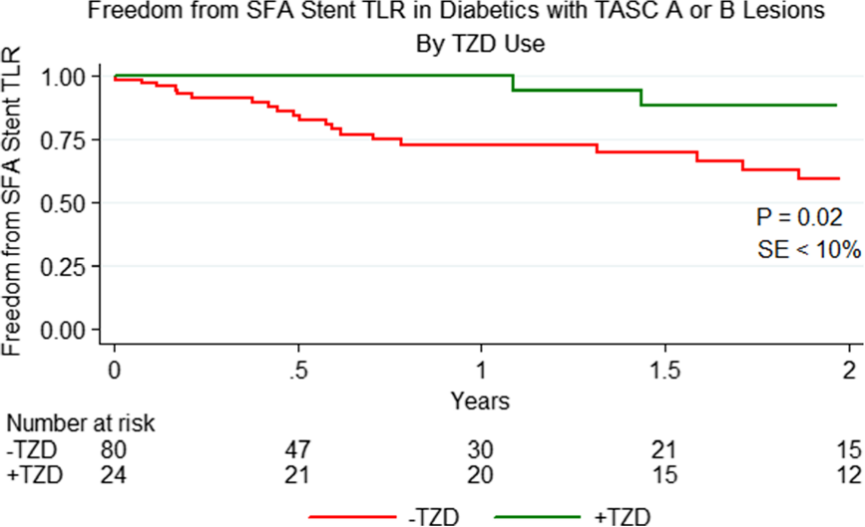
**Freedom from SFA Stent TLR among diabetics with TASC A or B Lesions by TZD use.** Freedom from TLR was 88.5% % for diabetics with TASC A or B lesions taking TZDs at the time of SFA stenting vs. 59.5% for those not taking a TZDs at 2 years. This difference was statistically significant, P = 0.02, with a standard error < 10% at all points on the graph.

To address the concern that disease severity was different for those patients taking TZDs vs. those patients not taking TZDs, we performed a second analysis limited to diabetic patients with TASC A and B lesions (Figure 1). This analysis excluded 27 patients with TASC C and D lesions who were not taking TZDs; therefore, 87 patients not taking TZDs and 24 patients taking TZDs were included in the cohort. Demographics, comorbidities, use of cardiovascular medications, and use of diabetic medications were similar for patients with TASC A and B lesions regardless of TZD use. However, the prevalence of CLI still remained lower for patients taking TZDs (Table 2). The mean stented vessel diameter was 5.4 mm (95% CI: 5.2-5.5 mm) for patients not taking TZDs vs. 5.5 mm (95% CI: 5.2-5.8 mm) for patients taking TZDs, p = 0.37. The mean stented length was 12.7 cm (95% CI: 11.0-14.3 cm) for patients not taking TZDs vs. 8.9 cm (95% CI: 6.6-11.2 cm) for patients taking TZDs, p = 0.02. Despite the exclusion of TASC C and D lesions, the association of TZD use with improved freedom from TLR remained: patients with a TASC A or B lesion taking a TZD at the time of SFA stenting had a significantly better outcome with 88.5% vs. 59.5% being free from TLR at 2 years, P = 0.02 (Figure 3).

**Table 2:** **Characteristics of diabetic patients with TASC A or B SFA lesions**

	+TXD N = 24	-TZD N = 87	P-value
Age	64.45	68.75	0.11
Male	79.17%	55.17%	**0.03**
HTN	97.67%	88.51%	0.66
HLD	79.17%	81.61%	0.78
CAD	54.17%	54.02%	0.99
CKD	17.39%	16.09%	0.88
COPD	4.17%	8.54%	0.48
Current smoker	12.50%	18.39%	0.50
Statin	62.50%	70.93	0.43
ASA	78.26%	82.72%	0.63
Plavix	39.13%	44.44%	0.65
Cilostazol	8.70%	13.58%	0.53
Insulin	50.00%	68.97%	0.10
Metformin	37.50%	31.03%	0.62
Sulfonylurea	27.17%	31.03%	0.81
CLI	33.33%	60.92%	**0.02**
Preop toe pressure	52.5	43.46	0.32
3 vessel outflow	41.67%	37.21%	0.69

Finally, in our multivariable model, we found that among all diabetic patients (n = 138), three variables were associated with freedom from TLR: CLI, insulin use and TZD use, with TZD use being protective (Table 3). However, the effect imparted by TZD use demonstrated only a trend towards significance in this pilot study.

**Table 3:** **Cox proportional hazards model**

	Hazard ratio	Standard error	P-value	95% CI
CLI	1.89	0.78	0.13	0.84-4.26
Insulin	1.87	0.81	0.15	0.79-4.39
TZD	0.33	0.21	0.08	0.09-1.13

Cox proportional hazards were performed to determine predictors of primary patency. Critical limb ischemia (CLI), insulin use, thiazolidinediones (TZD) use were identified as important variables.

## Discussion

The goal of this pilot, translational study was to identify if TZD use is associated with improved TLR in diabetics undergoing SFA stenting. This study is the first to show that diabetics taking TZDs at the time of primary SFA stenting have a lower reintervention rate within the first 2 years. In our small cohort, none of the patients taking TZDs had reinterventions within the first year; the 2 reinterventions which were performed occurred between 12 and 18 months following the initial procedure. Despite the mean stented length being slightly longer for patients with TASC A and B lesions not taking TZDs (12.7 cm vs 8.9 cm), this difference likely does not explain our result as it is clinically insignificant.

Although our Cox proportional hazards model only trended towards statistical significance, the model identified TZD use as a protective factor associated with improved TLR, supporting our Kaplan Meier analysis. The model also identified CLI and insulin use as predictors of worse freedom from TLR which is consistent with prior studies [2, 3]. The main limitations of our model were small cohort size and low number of failures. Despite these limitations, this model is likely reliable and should be tested in a larger cohort of patients.

The results from this study are consistent with data from the cardiology literature demonstrating that TZD use is protective against in-stent restenosis in the coronary circulation among diabetic patients [7–9]. Surprisingly, even when nondiabetic patients were randomized to TZD use vs. placebo, TZD use resulted in decreased in-stent restenosis following bare metal coronary stenting [10]. One unexpected finding in our study was that all diabetic patients taking TZDs appeared to have less severe disease as exemplified by the presence of only TASC A or B lesions and a lower prevalence of CLI as an indication for stenting. Emerging data suggest that TZD use may help modulate the development of atherosclerosis. Review of the coronary artery disease literature identified preliminary data showing decreased plaque burden following treatment with TZDs [11, 12]. However, it is unknown if the same is true for lower extremity atherosclerosis.

The mechanism through which TZDs protect against in-stent restenosis is an area of active study. TZDs are peroxisome proliferator-activatory receptor-gamma (PPAR-gamma) agonists and have many effects: insulin sensitization, anti-proliferation via blocking cell cycle regulators, anti-platelet aggregation, and inhibition of inflammatory cytokines such as tumor necrosis factor-alpha [13–16]. TZDs also enhance anti-inflammatory proteins such as adiponectin. Adiponectin is best known as an insulin sensitizing adipokine secreted from adipocytes. However, adiponectin is also secreted from VSMCs and affects VSMC phenotype which has implications for the development of in-stent restenosis [17].

VSMCs can exhibit two phenotypes: a proliferative phenotype, and a quiescent or nonproliferative phenotype [17]. In-stent restenosis develops after VSMCs adopt the proliferative phenotype allowing cells to proliferate and migrate inwards; this process is known as neointimal hyperplasia. We have demonstrated that adiponectin secretion by VSMCs promotes the nonproliferative phenotype of neighboring VSMCs in a paracrine manner [17]. Similarly, TZDs promote the nonproliferative VSMC phenotype via adiponectin secretion [18–20]. The protective effect of adiponectin against neointimal hyperplasia has also been demonstrated in animal models. Adiponectin knockout mice were shown to have increased neointimal hyperplasia following femoral artery wire injury versus wild type mice [21].

**Figure 4:**
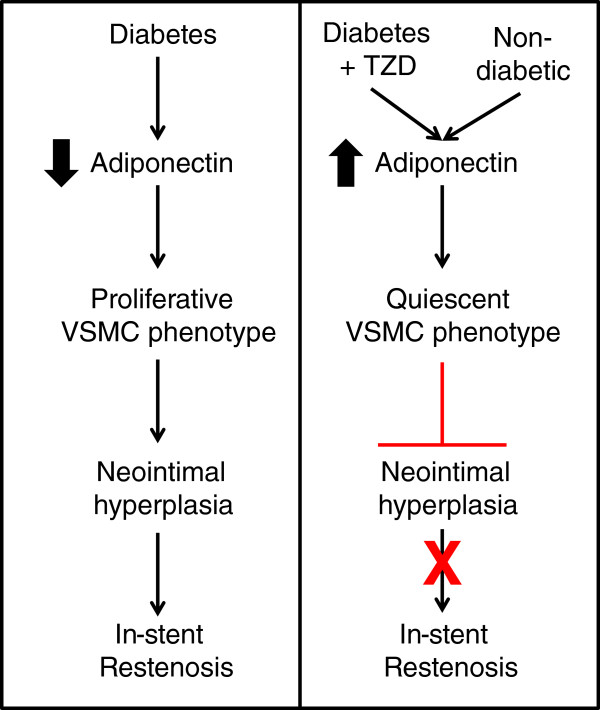
**Proposed hypothesis--in-stent restenosis, diabetes, thiazolidinedione use, adiponectin, and vascular smooth muscle cell phenotype.** The panel on the left proposes that diabetics are at increased risk for in-stent restenosis due to lower adiponectin levels increasing the likelihood that vascular smooth muscle cells (VSMCs) will adopt the proliferative phenotype. The panel on the right proposes that diabetics taking thiazolidinediones (TZDs) have higher adiponectin levels increasing the likelihood that vascular smooth muscle cells (VSMCs) will adopt the quiescent phenotype which decreases the chance of developing in-stent restenosis.

While specific mechanisms explaining the relationship between diabetes and propensity for in-stent restenosis have yet to be identified, the adiponectin pathway may be important as diabetics are known to have lower adiponectin levels than nondiabetics and higher rates of in-stent restenosis [22, 23]. We hypothesize that our results are secondary to higher adiponectin levels in patients taking TZDs (Figure 4). Support for this hypothesis is derived from previous studies demonstrating that low plasma adiponectin levels prospectively predicted in-stent restenosis for coronary bare metal stents [24–27]. In order to test this hypothesis further, we are currently enrolling patients in a prospective study designed to examine the ability of pre-procedure adiponectin levels to predict outcomes following lower extremity stenting. If serum adiponectin level predicts lower extremity in-stent restenosis, then a low adiponectin level may be used to identify patients who would benefit from treatment with a PPAR agonist, such as cilostazol and fibrates [28, 29].

Due to the retrospective nature of this study, several limitations exist. First, it is unknown why some patients were placed on TZDs and why others were not. The low use of TZDs may be due to changes in practice patterns related to the FDA’s recent black box warning regarding TZD use in patients with congestive heart failure [30]. Importantly, second generation TZDs are currently under development and have been reported to have a lower side effect profile [31]. Second, we were unable to control for hemoglobin A1c level; many of our patients receive primary care outside of our hospital system and therefore hemoglobin A1c was not consistently recorded in the medical record. Third, the study interval was relatively long which may raise concerns regarding changes in practice patterns. However, we evaluated TZD use and found it to be consistent across the study interval thereby decreasing this concern (data not shown).

## Conclusions

This small pilot study suggests that TZD use is associated with decreased reinterventions following SFA stenting in diabetic patients; this result should be investigated further using larger databases. Higher adiponectin levels resulting from PPAR-gamma activation secondary to TZD use may be a potential explanation for our results. We are prospectively collecting serum at the time of SFA stenting in order to measure adiponectin levels and evaluate this hypothesis. Other anti-inflammatory effects secondary to TZD use may also explain our results. We would like to strongly encourage national and regional quality improvement registries to collect information regarding patient’s specific diabetic regimens and use of PPAR agonists such as cilostazol and fibrates. Additional epidemiological data will help us better understand how to maximize medical management in diabetics undergoing endovascular intervention for peripheral arterial disease.

## Abbreviations

CLI: Critical limb ischemia
PPAR-gamma: Peroxisome proliferator-activatory receptor-gamma
SFA: Superficial femoral artery
TASC: TransAtlantic Inter-Society Consensus
TLR: Target lesion revascularization
TZD: Thiazolidinedione.
